# Ultracompact single-layer optical MEMS accelerometer based on evanescent coupling through silicon nanowaveguides

**DOI:** 10.1038/s41598-022-25760-8

**Published:** 2022-12-15

**Authors:** Chenguang Xin, Zhongyao Zhang, Xuhu Wang, Changjiang Fan, Mengwei Li

**Affiliations:** 1 School of Instrument and Electronics, North University of China, Taiyuan, 030051 China; 2 School of Instrument and Intelligence, North University of China, Taiyuan, 030051 China

**Keywords:** Optics and photonics, Applied optics

## Abstract

In this paper, a novel optical MEMS accelerometer is proposed based on evanescent coupling between parallel silicon nanowaveguides. The coupling length between nanowaveguides changes due to the input acceleration, leading to a great change of coupling efficiency. As a result, the applied acceleration can be obtained by measuring the transmission of waveguiding light. Simulation results with optical displacement sensing sensitivity of 32.83%/$\upmu$m within measurement range of 1.68 g is obtained. This design shows high compactness with no need of assembly, suggesting great potential in applications such as integrated photonic circuits.

## Introduction

IN past years, micro-electromechanical systems (MEMS) accelerometers have been attracting continuous attentions for its small size, low power consumption and easy incorporation with complementary metal–oxide–semiconductor transistor integrated circuits (CMOS IC)^1–3^. Several methods, including capacitive, piezo-resistive, piezo-electric, optical and so on^4^, have been developed to detect displacement of the proof mass introduced by input acceleration. Among these techniques, optical approaches have been demonstrated to be of higher precision and stability without suffering from electro-magnetic interference (EMI) at the same time^5,6^.

Benefitting from high precision for displacement measurement, grating interferometric structure and Fabry–Perot structure have been demonstrated as effective approaches to high-performance MEMS accelerometers^7–10^. Generally, a cavity structure consisting of two layers or end faces is necessary for effective optical interference, which typically requires a quite amount of space and relatively precise assembly^11,12^. Recently, efforts have been made to apply photonic crystal structure to accelerometers. Taking use of ultra-strong optical confinement properties, photonic crystal based MEMS accelerometers have been reported^13–15^. Since periodical structures are required, careful design and fabrication of waveguides or cavities are generally necessary in the case^16^. Considering the recent interest in developing integrated photonic devices and circuits, easily assembled MEMS accelerometers with ultracompact structures are in great demand. Silicon nanowaveguides offer an excellent platform for optical sensing. Strong evanescent field can be observed around nanowaveguides owing to tight optical confinement properties, which have already been used in applications such as supercontinuum source, resonators and couplers^17–19^.

In this paper, we initially demonstrate an ultracompact optical MEMS accelerometer through evanescent-field coupling between silicon nanowaveguides. The accelerometer, consisting of a proof mass, springs and silicon nanowaveguides, is designed on a single silicon-on-insulator (SOI) wafer. When input acceleration occurs, silicon nanowaveguide attached to the proof mass moves relatively to the silicon nanowaveguide fixed on the frame of the accelerometer. The coupling length between them changes accordingly, leading to a variation of coupling efficiency. Then, the input acceleration can be obtained by measuring output light power. Characteristics of this accelerometer are obtained with displacement sensing sensitivity of 32.83$\%$/$\upmu$m within measurement range of 1.68 g. The single-layer design results in a simple structure. In addition, the maximum coupling length within measurement range with a few micrometers between the nanowaveguides suggests that the sensor can be designed in ultra-compactness. The optical readout structure through the coupled waveguides requires a much smaller area than capacitive readout^20^. The results suggest great potential for the accelerometer in applications such as integrated inertial devices and circuits.

## Principle of the proposed optical MEMS accelerometer

**Figure 1:**
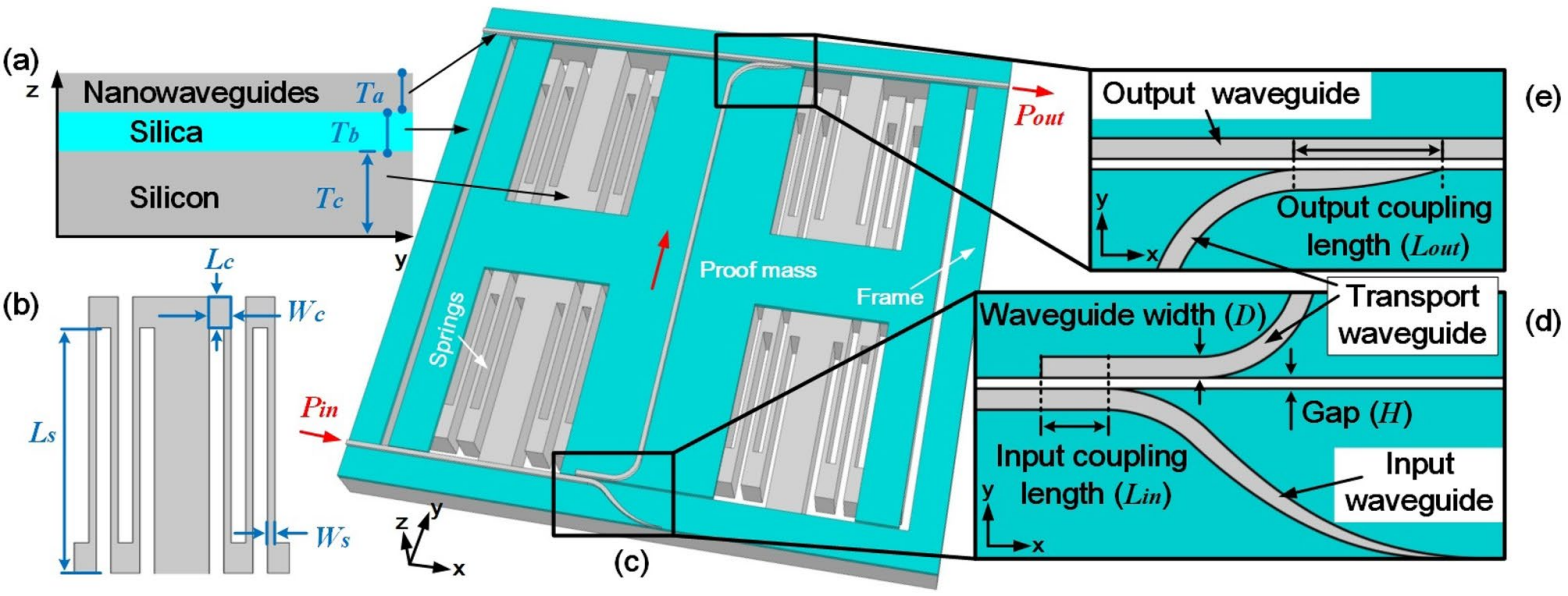
The structure of the proposed optical MEMS accelerometer. (**a**) Hierarchical design for SOI wafer. Constants $T_{a}, T_{b}$ and $T_{c}$ are thickness of the silicon nanowaveguides, silica substrate and proof mass. (**b**) Schematic of the spring. (**c**) Overall design of mechanical structure and optical path. (**d**) Input coupling structure. (**e**) Output coupling structure.

Our design consists of two parts: the optical displacement sensing part and the mechanical part, as shown in Fig. 1a and c. Specifically, the bottom layer is mechanical part, including serpentine springs (shown in Fig. 1b) and a proof mass. The silica layer is used as substrate. Three silicon nanowaveguides with the same section size [e.g., thickness ($T_{a}$) and waveguide width (*D*)] are located on the silicon layer. As seen in Fig. 1d and e, input waveguide and output waveguide are located on the frame, while the transport waveguide is located on the proof mass. Waveguides are arranged in parallel in sensing direction (*x* direction) with a certain coupling length ($L_{in}$ and $L_{out}$ respectively) and gap (*H*).

Both the input and the output waveguide are located along the edge of frame. The gap between the transport waveguide and other two waveguides is designed to be a constant in sub-wavelength scale. Firstly, light with power of $P_{in}$ is induced into the input waveguide. After propagating along the input waveguide for a certain distance, light is coupled into the transport waveguide through the input coupling structure as shown in Fig. 1d.

When there is no input acceleration, the light is coupled into bending transport nanowaveguide with input coupling efficiency ($\eta _{in}$) depending on the initial coupling length ($L_{0}$). After propagating along the transport waveguide across the proof mass, light is coupled into the output waveguide through the output coupling structure as shown in Fig. 1e. Depending on $L_{out}$, light in the transport waveguide is put out as the initial optical power ($P_{0}$) with the maximum output coupling efficiency ($\eta _{out}$). When there is an input acceleration (*da*), proof mass moves along the *x* axis, producing a certain relative displacement (*dx*). Transport waveguide located on the proof mass moves in *x* direction as well, resulting in a change of the input coupling length ($L_{in}$) expressed by Eq. (1).

1 $$\begin{aligned} L_{i n}=L_{0}+d x \end{aligned}$$

**Figure 2:**
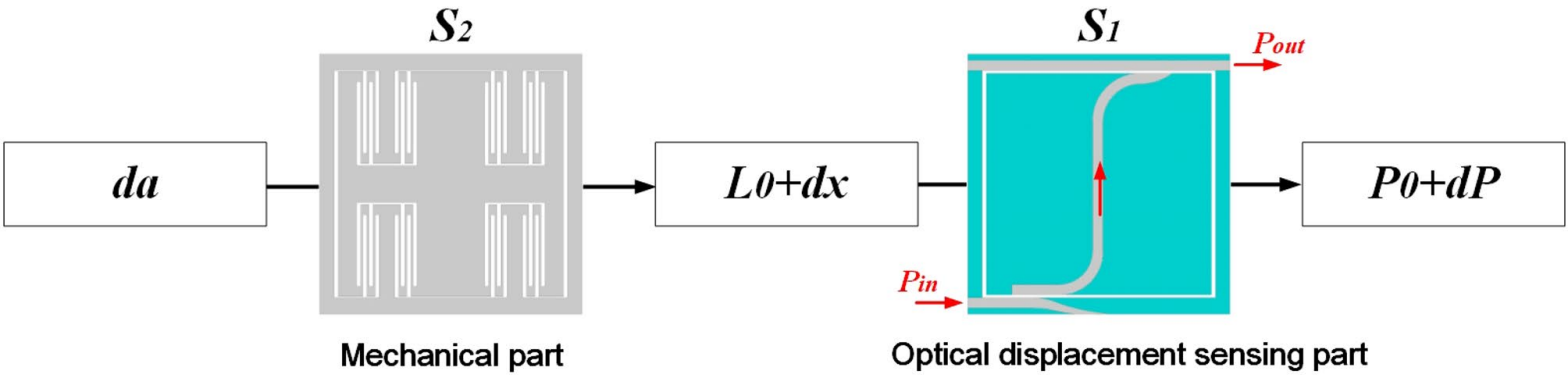
The transformation of input acceleration into output optical power.

$\eta _{in}$ from the input waveguide to the transport waveguide changes with $L_{in}$ simultaneously. In contrast to the input waveguide, the output waveguide is a straight waveguide across the whole frame. In this case, the output coupling length ($L_{out}$) as well as $\eta _{out}$ between the transport waveguide and the output waveguide remains a constant in spite of different locations of proof mass. As a result, the input acceleration can be derived from the output power ($P_{out}$) obtained at output port in output waveguide, which can be described as Eqs. (2) and (3).

2 $$\begin{aligned} P_{\textrm{out}}= & {} P_{0}+d P \end{aligned}$$

3 $$\begin{aligned} P_{\textrm{out}}= & {} P_{\textrm{in}} \cdot \eta _{\textrm{in}} \cdot \eta _{\textrm{out}} \end{aligned}$$

where *dP* is the increment of power due to input acceleration. $\eta _{in}$ is a variate related to the input acceleration, while $\eta _{out}$ is a constant in spite of external acceleration. The mentioned transformation of input acceleration into output optical power is shown in Fig. 2. The total sensitivity (*S*) and the total measurement range (*R*) of the optical MEMS accelerometer, revealing the relationship between input acceleration and output optical variation, can be given by Eqs. (4) and (5) respectively.

4 $$\begin{aligned} S= & {} S_{1} \cdot S_{2} \end{aligned}$$

5 $$\begin{aligned} R= & {} \frac{R_{1}}{S_{2}} \end{aligned}$$

where $S_{1}$ = *d*$\eta _{in}$/*d*$L_{in}$ is optical sensitivity, $S_{2}$ = *dx*/*da* is mechanical sensitivity and $R_{1}$ is measurement range of optical displacement sensing part.

## Design of the optical displacement sensing part

As mentioned above, the optical displacement sensing part mainly consists of three silicon nanowaveguides located on the silica layer. Benefitting from a high refractive index (e.g., 3.42 at 1550 nm), Silicon nanowaveguides show great optical confinement with geometry down to sub-wavelength scale. The strong evanescent field around the nanowaveguide offers an effective approach for optical coupling across different nanowaveguides^21,22^. The coupling efficiency is demonstrated highly relatively to parameters such as nanowaveguide size, gap between nanowaveguides and coupling length. An ultrahigh coupling efficiency over 95$\%$ has already been reported with careful setup of phase-matching conditions^23^.

**Figure 3:**
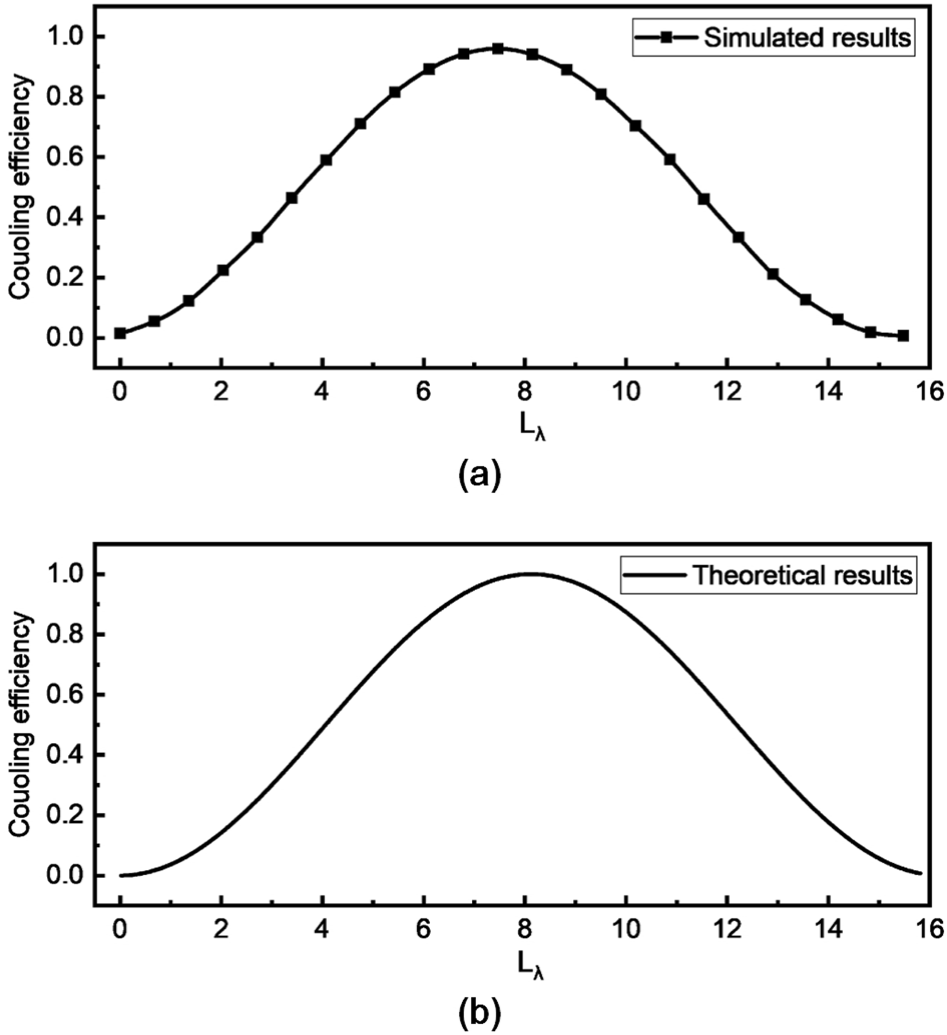
Comparison between (**a**) simulated results and (**b**) theoretical results. *D* is of 420 nm, *H* is of 100 nm, $\lambda$ is of 1550 nm and $\Delta n_{eff}$ is calculated to be of 0.06155.

In the input coupling structure as shown in Fig. 1d, $\eta _{in}$ is defined as the ratio of optical power in the input and transport waveguide. Coupling length dependent coupling efficiency can be expressed as^24,25^

6 $$\begin{aligned} \eta _{in}=\sin ^{2}\left( \frac{\pi \cdot \Delta n_{eff}}{\lambda } \cdot L_{in}\right) \end{aligned}$$

where $\Delta$ $n_{eff}$ is the difference of effective index between two supper modes existing in waveguides, which is related to geometries of waveguides. $\lambda$ is vacuum wavelength. Here, a normalized input coupling length in the unit of $\lambda$ is defined as $L_{\lambda }$ = $L_{in}$/$\lambda$ . In this case, Eq. (6) can be expressed as $\eta _{in}=\sin ^{2}\left( \pi \cdot \Delta n_{eff} \cdot L_{\lambda }\right)$. A comparison between simulated results and the theoretical results is shown in Fig. 3a and b respectively with *D* of 420 nm, *H* of 100 nm, $\lambda$ of 1550 nm and $\Delta n_{eff}$ calculated to be of 0.06155.

As shown in Fig. 3, simulated results and theoretical results have the same periodic tendency with period of about 16 $\upmu$m, showing a great agreement between them.

Equation (6) indicates a squared sinusoidal relationship between $\eta _{in}$ and $L_{in}$. However, for applications such as acceleration measurement, an approximately linear region is preferred. The optical sensitivity in our design is defined as changes in output power per unit displacement, depending on slope of coupling efficiency curve against coupling length. Simulation is carried out using finite-different time-domain (FDTD) method.

### The effect of end-face reflection

**Figure 4:**
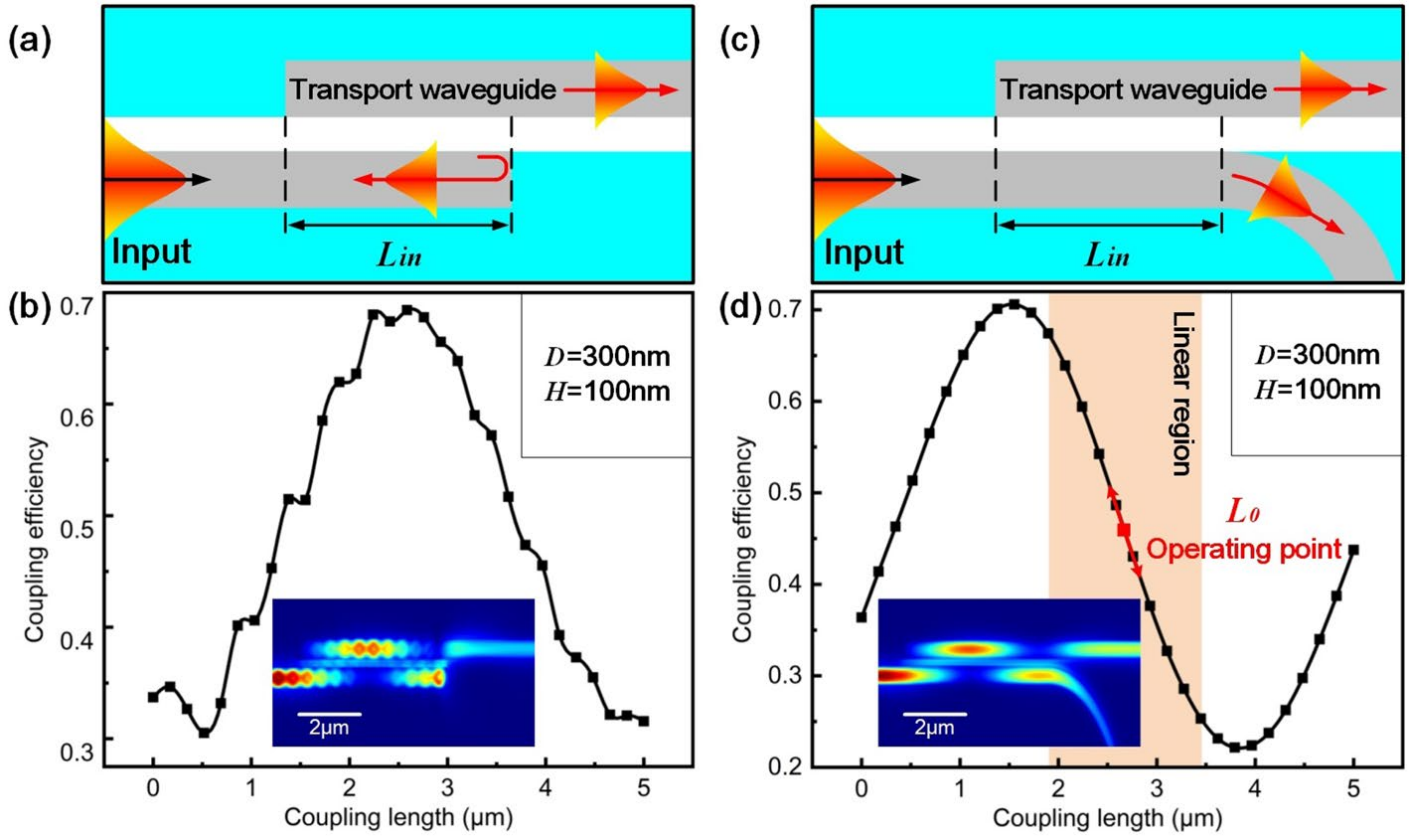
Simulation results of the effect of end-face reflection. (**a**) Coupling structure without pig tail and (**b**) corresponding simulated coupling efficiency. (**c**) Coupling structure with a pig tail at end of the input waveguide and (**d**) corresponding simulated coupling efficiency. In simulation, $T_{a}$, *D* and *H* is 220 nm, 300 nm and 100 nm respectively.

For typical coupling structure, two straight silicon nanowaveguides with rectangle end faces are parallelly located with a certain coupling length and gap (as shown in Fig. 4a). In simulation, $T_{a}$, *D* and *H* are 220 nm, 300 nm and 100 nm respectively. And the wavelength of input light is 1550 nm. Simulated coupling efficiency with $L_{in}$ changing from 0 to 5 $\upmu$m is shown in Fig. 4b. Obvious fluctuations are observed along the whole curve, which is believed to arise from interference between counter-propagating lights caused by end face effect of the input waveguide. As shown in the inset of Fig. 4b, interference patterns are observed along the waveguide.

To eliminate the effect of end-face reflection, an optimized model is proposed based on pig tail structure in Fig. 4c. The input waveguide consists of a straight waveguide and a bending pig tail. The end-face reflection is eliminated by using an absorbed perfectly matched layer (PML) boundary in Fig. 4c. Figure 4d shows the relationship between $\eta _{in}$ and $L_{in}$. A smooth curve is obtained with a relatively large slope within approximately linear region (indicated by the orange area in Fig. 4d). The inset confirms the elimination of endface-reflection-induced interference. In order to be operated in both the positive and negative directions, operating point is fixed in the middle of the linear region. When input acceleration is applied along the sensing axis in the positive direction ($+\,x$), the transport waveguide attached to the proof mass moves towards the negative direction ($-\,x$) relatively to the input waveguide. The increase of $L_{in}$ leads to a decrease in $\eta _{in}$. Similarly, when input acceleration occurs along the sensing axis in the negative direction ($-\,x$), $L_{in}$ decreases and $\eta _{in}$ increases. Thus, the input acceleration can be obtained from coupling length dependent coupling efficiency.

**Figure 5:**
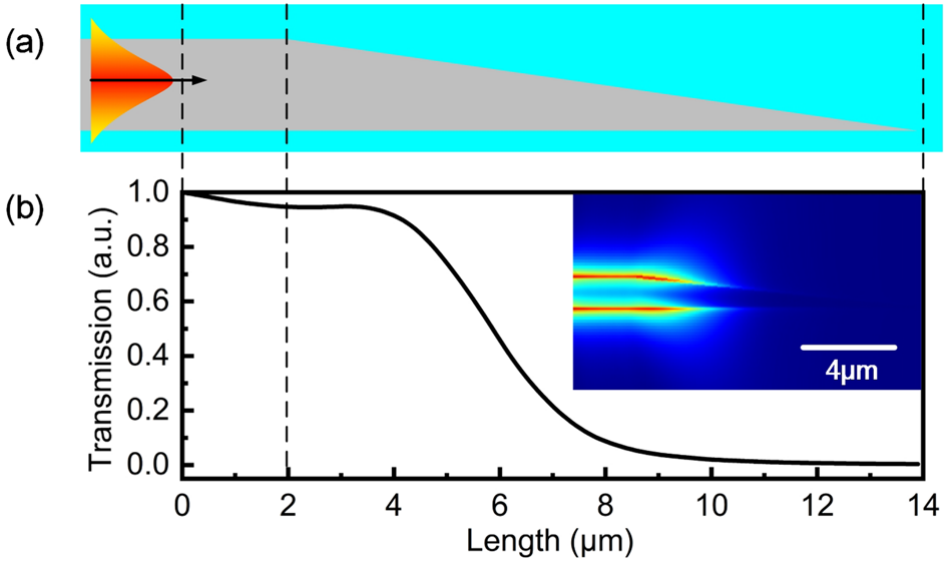
Optical propagating properties of pig tail. (**a**) Pig tail structure with a gradually decreased width started from 300 nm and a length of 12 $\upmu$m. (**b**) Transmission along pig tail. The inset is the spatial scattering patter in pig tail.

Optical propagating properties in pig tail are shown in Fig. 5. To demonstrate the effect of gradually decreasing architecture on optical loss, a simplified straight structure is used in simulation. With a length of 12 $\upmu$m and initial width of 300 nm (seen in Fig. 5a), strong optical scattering along the pig tail is observed resulting from decrease of geometry, leading to dramatically optical losses along the waveguide (seen in Fig. 5b). Most energy dissipate within a propagating length of 10 $\upmu$m. The power distribution along the pig tail shows a 100$\%$ loss of input power with reflection of 0$\%$ correspondingly, indicating a totally elimination of end-face reflection.

### The effect of waveguide geometries on optical sensitivity

**Figure 6:**
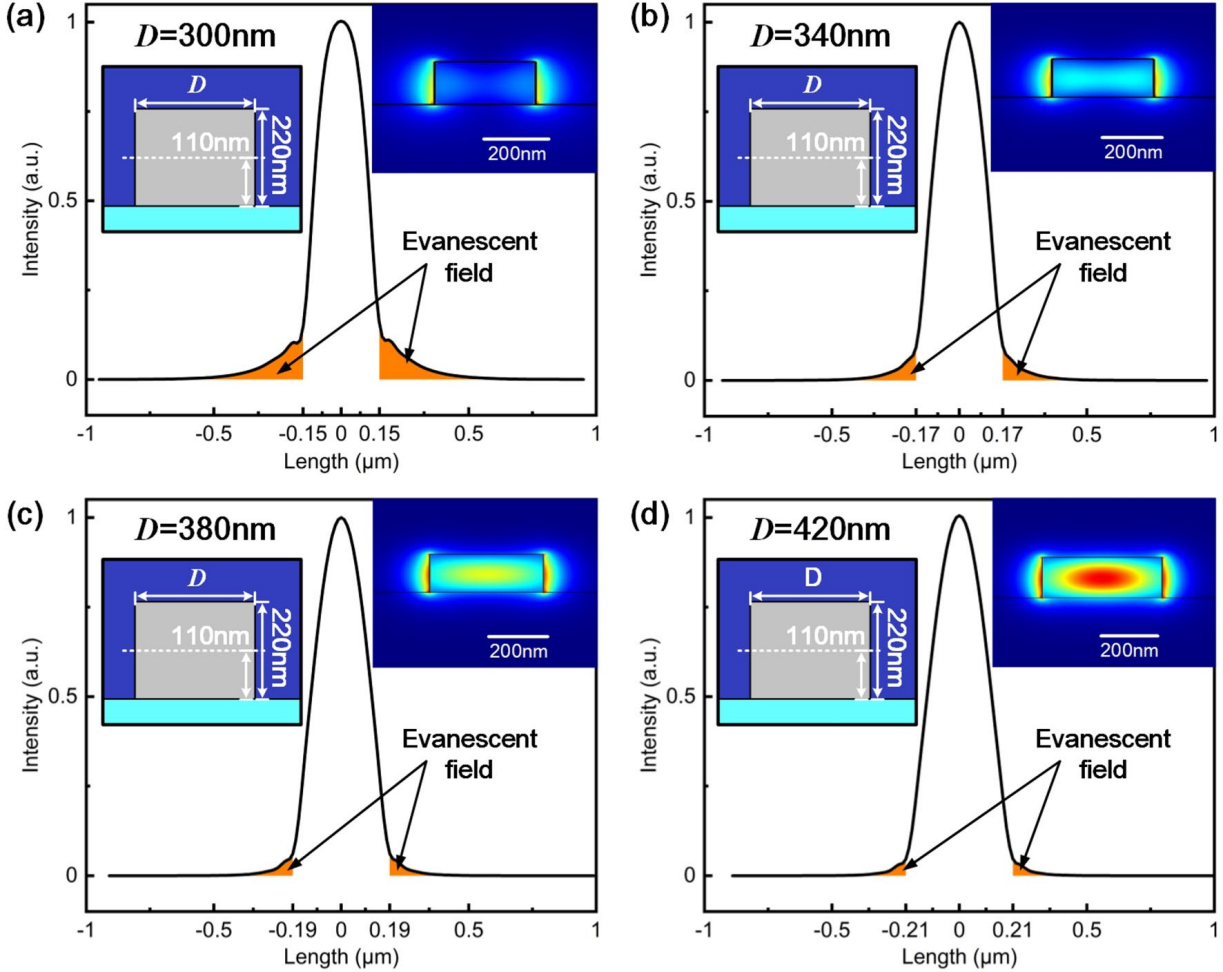
Profile of fundamental mode and intensity distribution in cross section for silicon nanowaveguides with different width of (**a**) 300 nm, (**b**) 340 nm, (**c**) 380 nm and (**d**) 420 nm respectively. Evanescent field is marked in orange. Insets on the left show cross sections of waveguides, in which the white dotted line indicates the positions of one-dimensional intensity distribution. Insets on the right show intensity profiles of fundamental mode.

First, the effect of waveguide width with a constant $T_{a}$ of 220 nm is studied. Figure 6 shows profiles of fundamental mode and one-dimensional intensity distribution in cross section for silicon nanowaveguides with different widths. The majority of light propagates inside waveguide. As *D* decreases from 420 to 300 nm, within the range of single mode operation, a stronger evanescent field is observed with power fraction increasing from 9.29 to 21.14$\%$, suggesting a larger modal overlap for a stronger coupling.

From Eq. (6), $\eta _{in}$ is a periodic function to $L_{in}$. Here, we define $L_{T}$ as the coupling length corresponding to a maximum coupling efficiency within the first coupling period. Detuning is defined as the difference between the actual coupling length and $L_{T}$.

7 $$\begin{aligned} \text { Detuning }=L_{\textrm{in}}-L_{T} \end{aligned}$$

**Figure 7:**
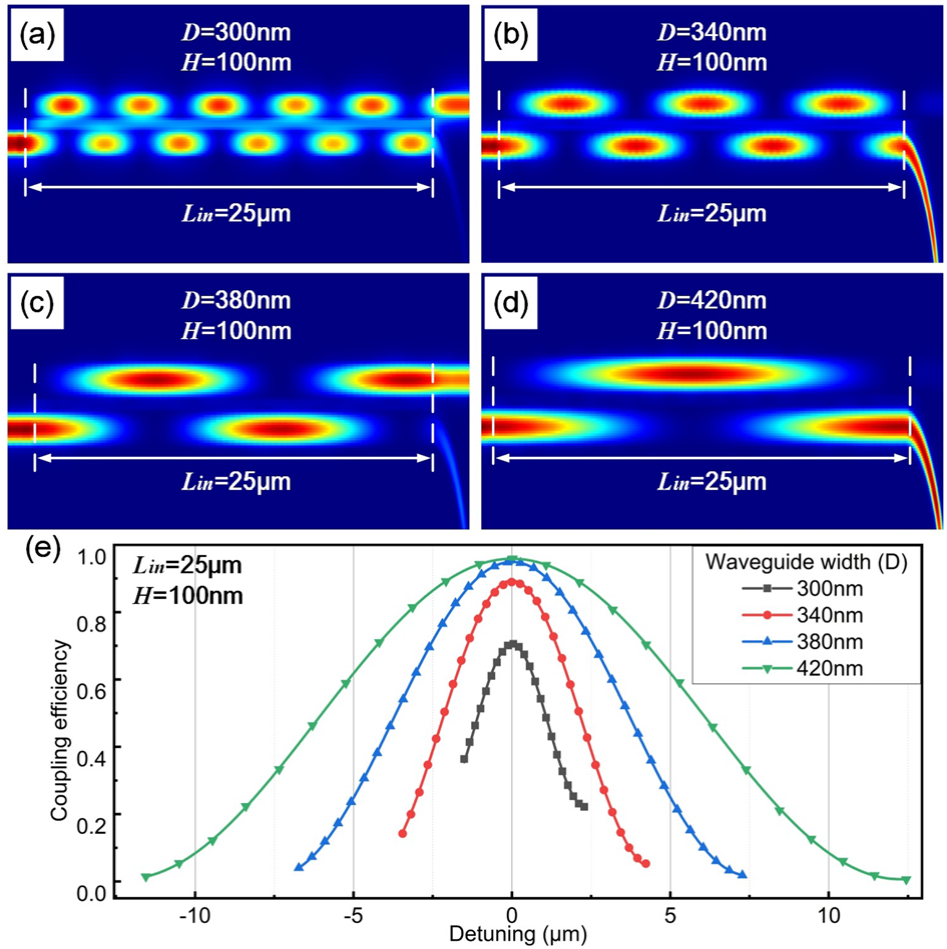
Power maps with different D of (**a**) 300 nm, (**b**) 340 nm, (**c**) 380 nm and (**d**) 420 nm respectively. In the simulation, $L_{in}$ is 25 $\upmu$m and *H* is 100 nm. (**e**) Coupling efficiency versus different detunings.

Figure 7a–d show power maps of the input coupling structure with *D* ranging from 300 to 420 nm. In the simulation, $L_{in}$ is 25 $\upmu$m and *H* is 100 nm. Power exchanges between two waveguides in a certain period. With a smaller *D*, there is a smaller period, which is explained by a stronger evanescent filed in such cases. For example, the period decreases from 24.00 to 4.48 $\upmu$m as *D* changing from 420 to 300 nm.

Moreover, relationship between coupling efficiency and detuning is presented in Fig. 7e. Detuning is swept within the first period with *D* of 300 nm, 340 nm, 380 nm and 420 nm respectively. Characteristics of the curves, such as slope and width of linear range (spectrum width with $R^{2}>0.99$), are listed in Table 1. With an increase of waveguide width, slope of falling edge goes down, while the linear range gets larger. For a higher optical sensitivity, *D* of 300 nm is adopted.

**Table 1:** Coupling properties with different waveguide widths.

Waveguide width (nm)	300	340	380	420
Slop ($\%/\upmu$m)	− 27.71	− 26.72	− 16.85	− 10.14
$R^{2}$	0.9920	0.9965	0.9957	0.9920
Linear range ($\upmu$m)	1.72	2.91	5.35	9.40
$L_{T}$ ($\upmu$m)	1.55	3.44	6.59	11.58

### The effect of gap width on coupling efficiency

**Figure 8:**
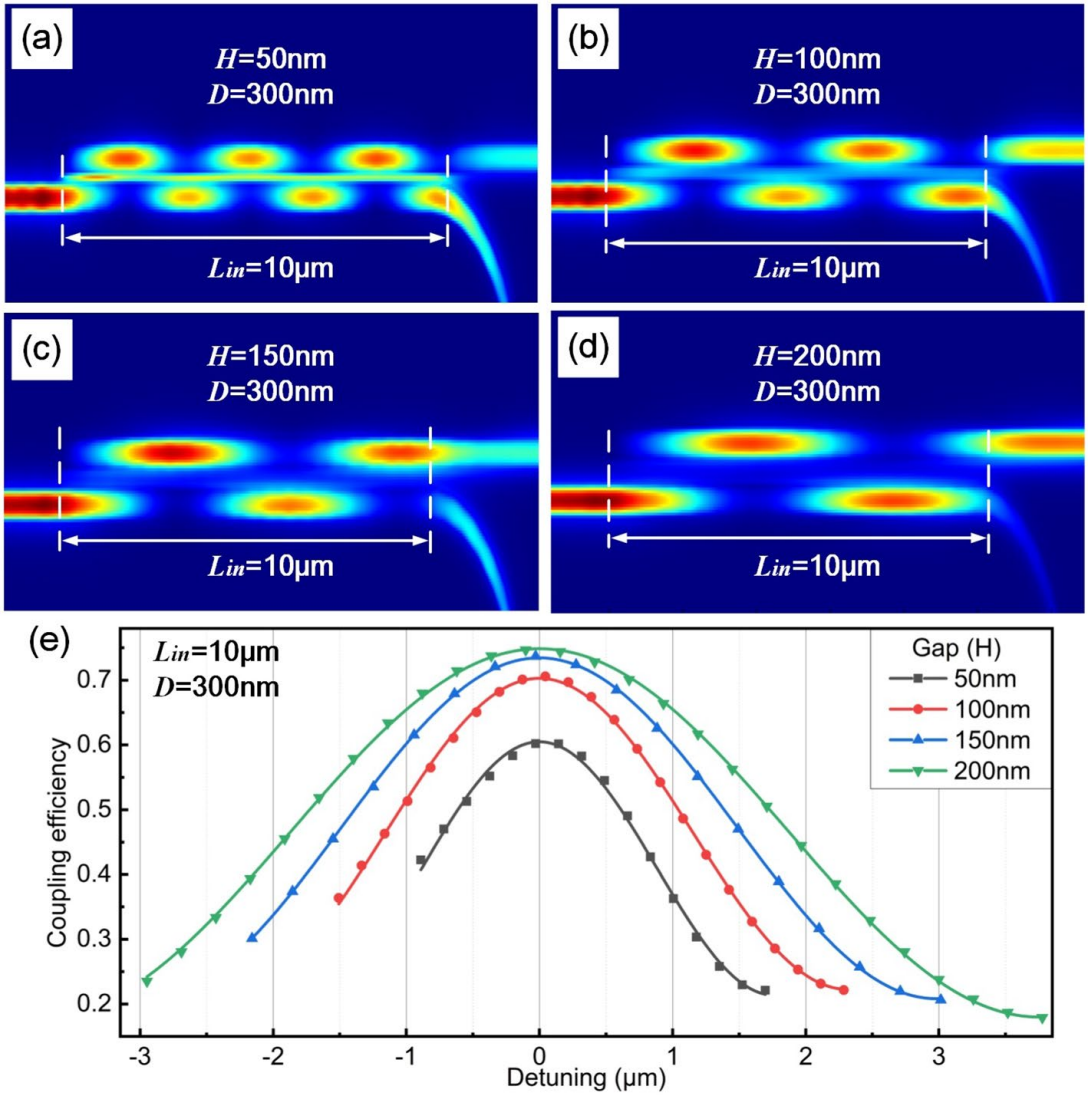
Power maps with different *H* of (**a**) 50 nm, (**b**) 100 nm, (**c**) 150 nm and (**d**) 200 nm respectively. In the simulation, $L_{in}$ is 10 $\upmu$m and *D* is 300 nm. (**e**) Coupling efficiency versus different detunings.

Similarly, with $L_{in}$ of 10 $\upmu$m and *D* of 300 nm, $\eta _{in}$ with different gap widths varying from 200 to 50 nm is investigated (as shown in Fig. 8). As gap width increases, period of power exchange becomes longer, leading to larger linear region and smaller slope. It is attributed to a less overlap between waveguiding modes. For example, the period decreases from 6.72 to 2.80 $\upmu$m as *H* changing from 200 to 50 nm.

As shown in Table 2, to obtain a higher optical sensitivity, *D* of 300 nm and *H* of 50 nm for silicon waveguides in optical displacement sensing part is adopted, corresponding to an optical sensitivity of 32.83$\%$/$\upmu$m within linear range of 1.04 $\upmu$m.

**Table 2:** Coupling properties with different gap widths.

Waveguide width (nm)	50	100	150	200
Slop ($\%/\upmu$m)	− 32.83	− 28.65	− 22.34	− 19.14
$R^{2}$	0.9957	0.9964	0.9927	0.9917
Linear range ($\upmu$m)	1.04	1.55	2.44	3.10
$L_{T}$ ($\upmu$m)	0.87	1.55	2.13	2.84

## Design of the mechanical part

**Figure 9:**
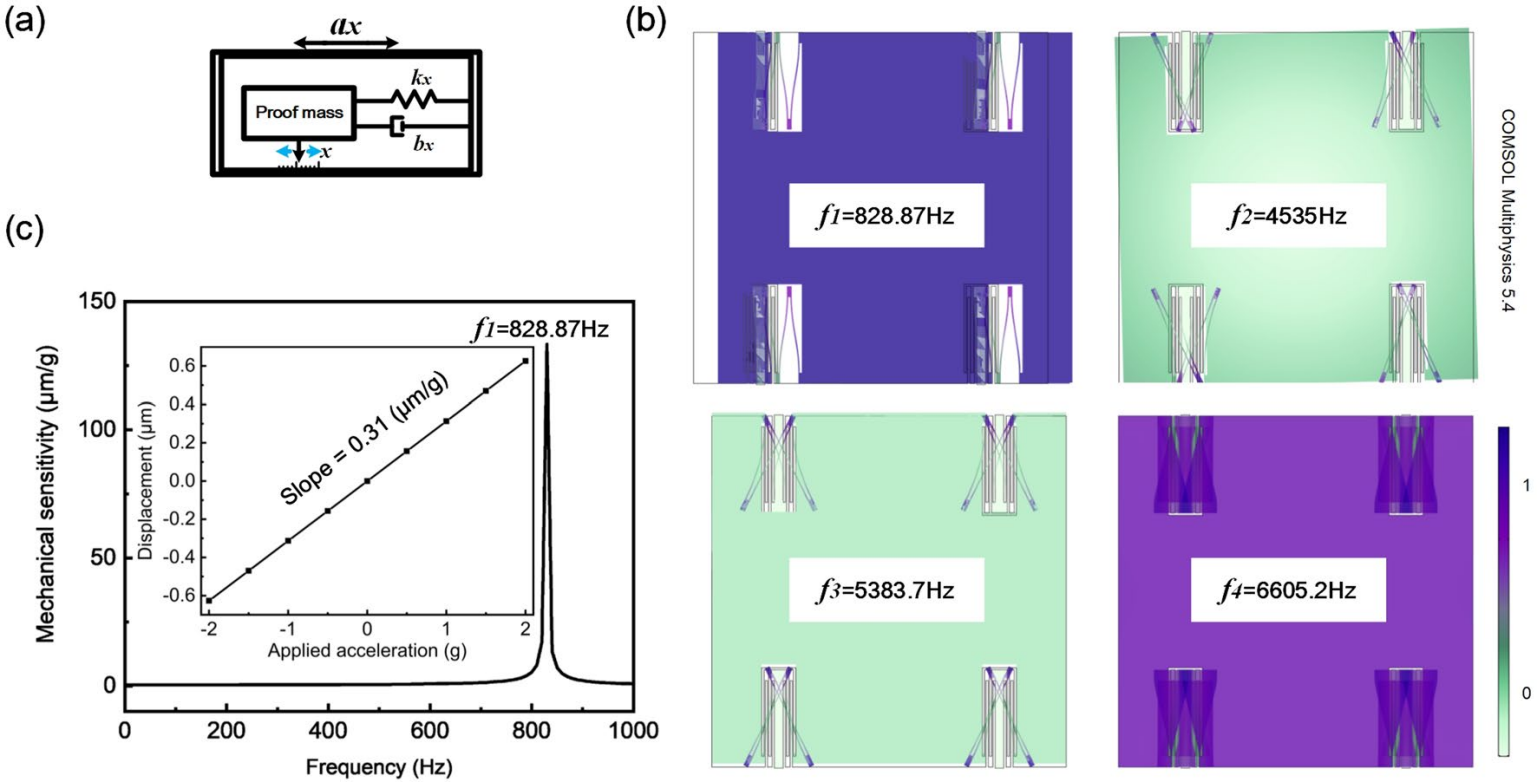
Mechanical characteristics of the proposed accelerometer. (**a**) The lumped model. (**b**) The first four resonance modes. (**c**) Frequency response, the inset shows mechanical sensitivity.

The mechanical part of the proposed accelerometer can be described as a mass-spring-damper mechanical model (seen in Fig. 9a) illustrated by a second order differential equation. Mechanical sensitivity can be given by^26^.

8 $$\begin{aligned}{} & {} \frac{x(\omega )}{a(\omega )} \approx \frac{1}{\omega _{m}^{2}}\left( \omega \ll \omega _{m}\right) \end{aligned}$$

9 $$\begin{aligned}{} & {} \omega _{m}=2 \pi \sqrt{\frac{k}{m}}, \end{aligned}$$

where $\omega _{m}$ is the resonance frequency, relating to spring constant *k* and mass *m*. From equations above, the mechanical sensitivity can be promoted by using a heavier proof mass. To install the four springs inside the proof mass can greatly increase the compactness of whole structure^27,28^. Serpentine springs are designed symmetrically connecting to the frame. Physical and geometrical parameters of the accelerometer are shown Table 3, including dimensions of springs shown in Fig. 1b.

**Table 3:** Physical and geometrical parameters of the accelerometer.

Symbols	Parameter	Value
*E*	Young’s modulus of silicon	170 GPa
$\rho$	Density of silicon	2329 $kg/m^{3}$
r	Poisson ratio of silicon	0.28
$T_{a}$	Thickness of silicon nanowaveguides	220 nm
$T_{b}$	Thickness of silica substrate	3 $\upmu$m
$T_{c}$	Thickness of proof mass	300 $\upmu$m
*L W*	Size of proof mass	5237 $\upmu$m 5237 $\upmu$m
$L_{s}$ $W_{s}$	Size of span beam	1287 $\upmu$m 17 $\upmu$m
$L_{c}$ $W_{c}$	Size of connector beam	161 $\upmu$m 51 $\upmu$m
k	Spring constant	306.28 N/m
m	Mass	17.60 mg

Figure 9b shows the first four resonance frequencies. Generally, accelerator is operated within the first resonance frequency ($f_{1}$ = 828.87 Hz). The second resonance frequency is much larger than the first one, indicating that other modes do not affect the measurement operation. Equation (9) shows that the mechanical sensitivity is a constant, provided that the operating frequency is much less than the resonance frequency. As shown in Fig. 9c, the working band width of the proposed accelerometer is about 700 Hz within a relatively flat frequency response.

Using parameters shown in Table 3, mechanical sensitivity is investigated in theory. As shown in Fig. 9c, a linear relationship is obtained for displacement within an applied acceleration range from − 2 to $+$ 2 g. The result shows a mechanical sensitivity of 0.31 $\upmu$m/g, approximately the calculated value in Eq. (9).

As a result, nanowaveguides with section size of 300 nm 220 nm and gap width of 50 nm are adopted for displacement measurement resulting in $S_{1}$ of 32.83$\%/\upmu$m and $S_{2}$ of 0.31 $\upmu$m/g. The relatively high total sensitivity (*S*) of the optical MEMS accelerometer is calculated to be 10.18$\%$/g given by Eq. (4). In addition, the total measurement range (*R*) of the accelerometer can be obtained by Eq. (5) as 3.36 g, limited by a linear region of optical displacement measurement with 1.04-$\upmu$m width. Considering the accelerometer is operated in both positive and negative direction, the measurement range of the accelerometer is 1.68 g.

**Figure 10:**
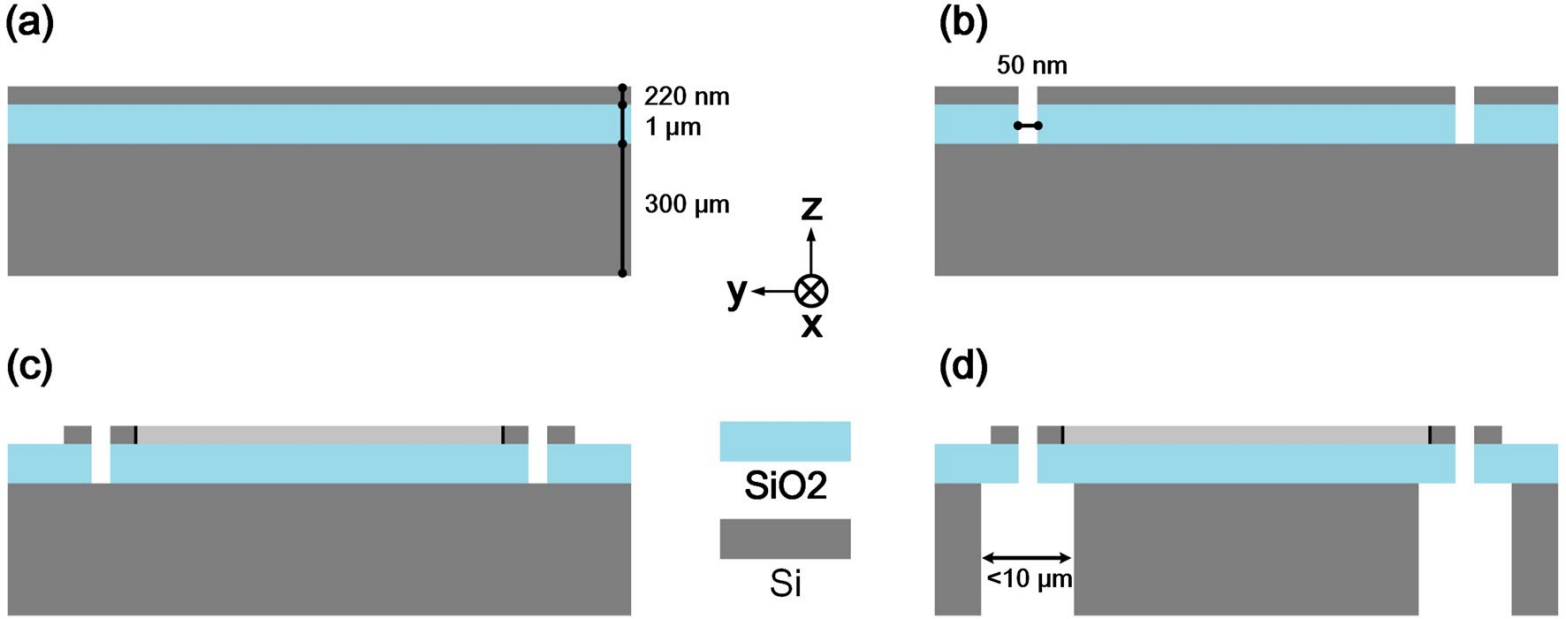
Simplified process fabrication flow. (**a**) SOI-wafer. (**b**) Top layer as well as the oxide layer is etched by RIE leading to 50 nm wide gaps along the sensing axis. (**c**) Silicon nanowaveguides are fabricated through RIE. (**d**) Mechanical part including springs and proof mass is fabricated by DRIE, which can keep the gap between proof mass and frame less than 10 $\upmu$m.

For a thick layer (of 300 $\upmu$m) is needed for a large mechanical sensitivity, the gap between proof mass and frame in micromechanical structure is usually larger than 2 or 3 $\upmu$m, while sub-wavelength-scale gap between silicon nanowaveguides is necessary for evanescent coupling. In this case, a simplified process fabrication flow is proposed shown in Fig. 10a–d. As illustrated in Fig. 1a and Table 3, the thickness of the top layer, oxide layer and the bottom layer in SOI wafer is 220 nm, 1 $\upmu$m and 300 $\upmu$m respectively, as marked in Fig. 10a. In Fig. 10b, by using reactive ion etching (RIE) technique, top layer as well as the oxide layer is etched, producing 50 nm wide gaps along the sensing axis. In Fig. 10c, three silicon nanowaveguides are fabricated through RIE. A main challenge for the attenuation in the nanowaveguides is the surface roughness. However, several low-loss process methods have been developed to deal with the problem^29–31^. Given an optimized process method, about 1-nm line-edge roughness induces transmission loss of 1 dB/cm for 300 nm wide waveguides. In our design, about total 99.7$\%$ incident power is kept in nanowaveguides for transmission and detection. In Fig. 10d, springs and proof mass are fabricated by using back-sided deep reactive ion etching (DRIE) technique, which can keep the gap between proof mass and frame less than 10 $\upmu$m. This design solves the incompatibility in geometries between MEMS structure and silicon nanowaveguides with reasonable aspect ratio in fabrication^32–34^.

## Conclusion

In this paper, a novel optical MEMS accelerometer with optical sensitivity of 32.83$\%/\upmu$m within 1.68 g has been presented. The accelerometer is proposed based on evanescent-field coupling between three silicon nanowaveguides. Located on proof mass and frame respectively, these nanowaveguides overlap with each other in the sensing direction with a constant gap in sub-wavelength scale. When input acceleration occurs, coupling length between the nanowaveguides changes resulting from relative displacement caused by proof mass, leading to a change in coupling efficiency. As a result, the acceleration can be obtained by measuring the total transmission. Both the optical and mechanical features of the proposed accelerometer have been investigated in simulation. A section size of 300 nm 220 nm for the nanowaveguides and gap between proof mass and frame of 50 nm are decided. Benefitting from an ultracompact single-layer structure, we believe the design show great potential in integrated optical inertial devices and circuits.

## Data Availability

Data underlying the results presented in this paper are not publicly available at this time but may be obtained from the corresponding auther upon reasonable request.
